# Dorsal Nasal Skin Necrosis Following Rhinoplasty in Patients Treated with Topical Retinoids

**DOI:** 10.1055/s-0045-1804533

**Published:** 2025-03-10

**Authors:** Ian Shyaka, Natasha Natasha, Anjana Elangovan, Karthik Ramasamy

**Affiliations:** 1 Department of Aesthetic Plastic Surgery, Chennai Plastic Surgery, Chetpet, Chennai, Tamil Nadu, India

**Keywords:** rhinoplasty, dorsal nose skin necrosis, tretinoin

## Abstract

Rhinoplasty is one of the most performed procedures by plastic surgeons worldwide, for both functional and cosmetic indications. The demand for aesthetic rhinoplasty in India has been on the rise over the past decade. Despite this rise in demand, rhinoplasty is still considered one of the most technically demanding procedures in plastic surgery. Complications following rhinoplasty are reported to be 4 to 18.8%, with dorsal skin necrosis being a rare complication that occurs in less than 1% cases. Out of a total of 244 rhinoplasties performed by a single surgeon at our institution, 2 cases of dorsal skin necrosis were encountered (0.8%). Currently, there is no literature describing dorsal skin necrosis following rhinoplasty among the Indian population. We present two patients who experienced dorsal nasal skin necrosis following open rhinoplasty while on topical 0.05% tretinoin treatment and highlight our management approach.

## Introduction

Rhinoplasty is one of the most commonly performed procedures by plastic surgeons worldwide. 1 . Rhinoplasty complications can arise even for experienced surgeons, with complication rates ranging between 4 and 18.8%. 2 Complications following rhinoplasty can be classified as hemorrhagic, infection, traumatic, functional, and aesthetic. While the majority of these do not pose as a life-threatening condition, major life-threatening complications such as rhinorrhea, pneumothorax, and subarachnoid hemorrhage are rarely encountered. 3 Dorsal nasal skin necrosis postrhinoplasty is reported in less than 1% cases. 4 In this report, we discuss two patients who experienced dorsal nasal skin necrosis following aesthetic rhinoplasty.

**Patient 1:** The first patient is a 32-year-old woman on topical tretinoin 0.05% for facial acne treatment for 7 months. She underwent open rhinoplasty with cephalic trimming, minimal defatting of the bulbous tip, and bilateral alar base reduction. Tip augmentation was performed using a columella cartilage strut harvested from the nasal septum. Bilateral lateral nasal bone osteotomies were performed, and diced cartilage was placed over the nasal dorsum using a syringe. Steri-Strips were applied on the dorsal nose and an aluminum Denver splint was safely applied. Upon removing the splint 1 week postoperatively, full-thickness skin loss over the dorsal nose was observed, measuring 3 × 1 cm ^2^ .

**Fig. 1:**
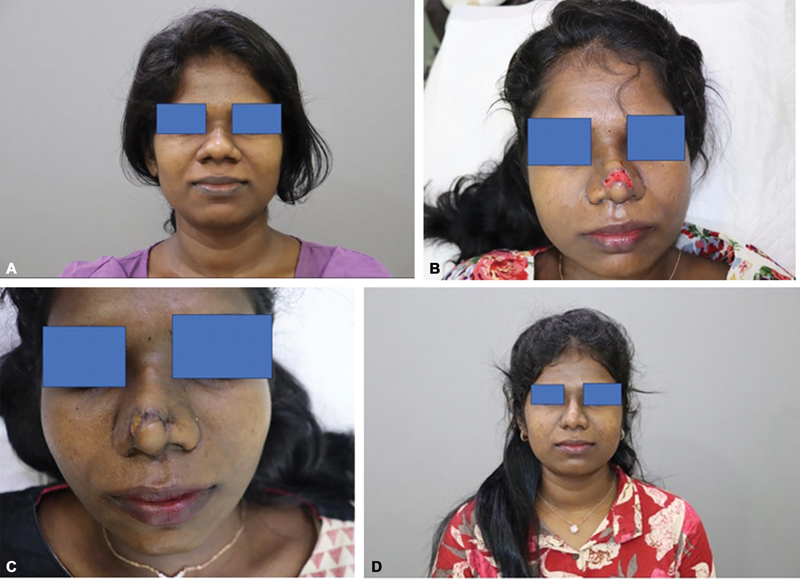
( **A** ) Patient preoperative picture. ( **B** ) At 1 week postoperatively. ( **C** ) Local tissue advancement to minimize wound surface. ( **D** ) At 1 year postoperatively. The patient after complete wound healing and two sessions of dermal fillers to correct residual deformities.

Gentle debridement of the necrotic skin was performed, and local tissue advancement was performed to downsize the wound surface. The wound was managed conservatively with dressing changes. Six months after the wound healing, she received two sessions of dermal hyaluronic acid filler injections to correct residual deformities, and an aesthetically acceptable result was eventually obtained ( Fig. 1 ).

**Fig. 2:**
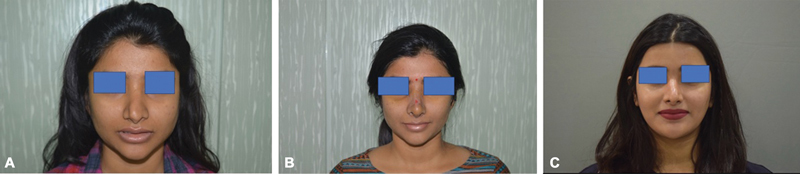
( **A** ) Pre-op. ( **B** ) At 1 week post-op. ( **C** ) At the 1-year follow-up.

**Patient 2:** A 34-year-old woman presented with a crooked nose following rhinoplasty 2 years ago. She had been using topical 0.05% tretinoin for acne for 6 months. We performed a secondary open rhinoplasty with bilateral lateral osteotomies, spreader grafts, and a columellar strut using a septal cartilage graft. Diced cartilage placement over the nasal dorsum was done, and an aluminum Denver splint applied postoperatively. One week later, she developed partial-thickness dorsal skin necrosis, which was managed nonsurgically with dressing changes. Six months after the wound had healed, she underwent facial carbon dioxide laser resurfacing and a touch-up session with hyaluronic acid fillers ( Fig. 2 ).

## Discussion

Complications postrhinoplasty are very stressful for both the patient and the surgeon. Although rarely reported, skin necrosis postrhinoplasty can present as superficial or full-thickness skin loss. Typically, superficial necrosis occurs due to overly tight bandaging or dressing. These cases are usually managed conservatively, and if necessary, revision procedures can be performed after the scar has matured. Full-thickness skin necrosis, on the other hand, typically necessitates secondary surgery and is associated with much less satisfactory outcomes. Smoking is a known risk factor for skin necrosis postsurgery, 5 but none of our patients were smokers. Both our patients reported were on topical 0.05% tretinoin for acne treatment and we queried if this had a role in the complications. Since the U.S. Food and Drug Administration (FDA) approved tretinoin in 1971, retinoids alone or combined with other agents have become the mainstay of acne treatment. 6 Isotretinoin, an oral retinoid, is also commonly used to treat inflammatory skin conditions, genodermatoses, skin cancer, and other skin disorders. This is due to its anti-inflammatory, immunomodulatory, and antineoplastic properties. 7 Low-dose isotretinoin has been safely utilized as an adjunct in rhinoplasty to enhance skin thinning and improve outcomes. Yahyavi et al demonstrated that oral isotretinoin 20 mg daily given for 2 weeks before surgery and up to 2 months following the surgery was safe and associated with better aesthetic outcomes. 8 Additionally, Blough et al reported starting oral isotretinoin, 20 mg daily, as early as 1 week to 2 months before surgery. 9 However, the use of isotretinoin before rhinoplasty surgery still has many controversies 9 as there are no universal criteria regarding the duration or dosage. Currently, there are limited data on the safety of topical retinoids prior to rhinoplasty. In patient 1, bilateral alar base reduction and open rhinoplasty were performed. In both patients, nasal dorsal dissection was performed in a loose areolar tissue plane on the cartilaginous vault and the subperiosteal plane on the bony vault with minimal trimming of the fibrofatty tissues performed.

The senior author has performed simultaneous alar base reductions and open rhinoplasty over the years with no such complications; however, we still queried if this combination was a contributing factor in patient 1. Simultaneous alar base reduction and open rhinoplasty have been proven to be safe over the years. 10

Although dealing with these complications postrhinoplasty can be very frustrating to both the patient and the surgeon, it is crucial to refrain from being surgically aggressive in the early healing phase. Multiple surgeries might worsen the outcome or create a more distorted anatomy. Nonsurgical options such as dermal fillers and laser therapy can be safely utilized to achieve aesthetically pleasing results. However, we recommend utilizing these modalities at least 6 months after the external wounds have resolved.

While we are unable to provide definite recommendations, we believe this report raises important considerations that merit further investigation. There may be a need to study the long-term use of topical retinoids prior to rhinoplasty. Alternatively, enhanced precautionary measures should be considered for patients undergoing rhinoplasty while on long-term topical retinoid therapy.

Based on this experience, we routinely advise patients to discontinue topical retinoid use for at least 6 weeks before rhinoplasty. Additionally, it is crucial to thoroughly review a patient's current topical regimens to exclude retinoids, as this was discovered later in the cases reported above. However, we acknowledge the need for further studies with larger patient populations or multicenter studies to evaluate the safety of topical retinoids in rhinoplasty and draw more reliable conclusions.

Nonsurgical aesthetic techniques, such as dermal fillers, and lasers serve as valuable adjuncts in the management of postrhinoplasty complications and whenever suitable should be considered as an alternative approach without additional surgery.
